# Analogy of Chronic Hepatitis and Phthisis Pulmonalis

**Published:** 1817-09

**Authors:** 


### *For the London Medical and Physical Journal.*

### *Analogy of Chronic Hepatitis and Phthisis Pulmonalis.*

IN perusing your Journal of July, I was much gratified by observing that so eminent a practitioner as Dr. Kinglake has at length called the attention of the medical profession to the similarity which exists between Phthysis Pulmonalis and Chronic Hepatitis.

The interesting period of life at which phthysis usually occurs, and the still more interesting objects who are daily falling victims to its baneful effects, must inspire every one with a wish to elucidate, as much as possible, its real nature and cause, and to point out a mode of treatment founded on rational principles. With this view, I shall relate two cases, out of many which have fallen under my notice, in which the pulmonary affection appeared to be connected with a disordered state of the abdominal viscera in general, and of the hepatic system in particular. During the early part of last January, I was requested to visit George Davis, a child about three years of age. He had been labouring under a pulmonary affection for two months, and was now, as the mother thought, in a deep decline. Indeed, the appearance of the child seemed to warrant such an opinion: he was much emaciated, had a constant cough, difficult respiration, a small quick pulse, and profuse perspirations during the night. The medical treatment had hitherto been entirely directed to mitigate these symptoms: leeches and blisters had been frequently applied to the thorax; a mixture given to relieve the cough, together with emetics to unload the air-vessels of that quantity of mucus which was always collecting in them. Under this treatment the child seemed rapidly declining. Upon inquiring the state of the bowels, the mother told me they were quite regular; but, upon asking more particularly about the nature of the evacuations, I found they were at all times exceedingly unnatural and offensive. The abdomen was not much swollen, but hard, and painful on pressure. The child frequently complained of pain in the head; the eyes, however, retained their healthy appearance.

Under these circumstances, I thought the best treatment I could adopt would be to remove from the lungs that additional irritation which must necessarily be caused by the present disordered state of the abdominal viscera; and prescribed 1 grain of calomel with 3 grains of chalk to be given every night and morning, with a mixture, to abate the urgency of the the cough. In the course of a week I again saw the child. The powders had been taken regularly, but so much difficulty had been experienced in giving the mixture that it was omitted after the first day. During the first two or three days there was no perceptible amendment of the fæcal discharges, but since that time they had become more natural; the cough was also less troublesome: the powders were continued. Under this treatment, the evacuations gradually acquired a healthy appearance; and, in the course of a fortnight, there remained no vestige of pulmonary disease. Small doses of calomel and jalap were persisted in during another month, at which time the child enjoyed better health than at any former period of his life.

It perhaps may be said that this was a case of Tabes Mesenterica: I grant it might be, and I relate it merely to shew how dependent the pulmonary disorder was upon that of the abdominal viscera. In many similar cases which occurred subsequently, I directed my sole attention to the state of the bowels, and invariably found the pulmonary affection to subside in proportion as the digestive organs resumed their proper functions.

During my attendance on the fore-mentioned case, I was requested to see George Glenny, aged thirty years, of a delicate habit, and by trade a white-smith. He had been affected with a troublesome cough for three or four months, and, during the last, had been subject to frequent expectoration of blood: this had now ceased, and was succeeded by a copious purulent expectoration of a fawn colour. His cough was very urgent; respiration difficult, accompanied by an almost insufferable sense of tightness across the thorax; pulse quick and small; weakness so great that he was scarcely able to walk across the room; and the night-sweats more profuse than I had ever known them. His bowels were habitually costive, but, during the last fortnight, this costiveness had alternated with diarrhœa; the evacuations were unhealthy in appearance. As he was gradually growing worse under a treatment directed to the pulmonary affection, I thought the plan instituted in the former case might be of service in this, and gave him alterative doses of calomel, with mild aperient medicines;—he took also the nitrous acid mixture, and lived chiefly on a milk diet.

In the space of a fortnight, the bowels became regular and the discharges healthy; the cough and night-sweats had nearly left him; and he was able to walk out for an hour in the day. He still complained much of tightness across the chest and pain in the side, which were relieved by the application of a blister and small doses of digitalis.

At

At the end of a month, debility seemed to be his only ailment; he was therefore sent into the country. After three weeks' stay, he returned to town to resume his former occupations, enjoying as good a state of health as he had ever possessed.

In this case it must be allowed the pulmonary affection was entirely a secondary disease; yet, had it been suffered to proceed, would it not have become independent of its primary cause, and have terminated in phthisis?

The cases mentioned by Dr. Kinglake are considered by him to have been cases of chronic hepatitis mistaken for phthysis; and this opinion is entertained because the pulmonary affection ceased as soon as the hepatic disorder was subdued: yet these cases so resembled phthysis as to have induced a treatment appropriate to that disease; and, I ask, would not the pulmonary disorder have become established, unless it had been interrupted in its progress by the judicious treatment of Dr. Kinglake?

That idiopathic phthysis does often occur, cannot be denied; but, in my opinion, this disease may be, and is, frequently induced by disorder of the abdominal viscera. It may be argued that examination of phthysical subjects has commonly shewn a disorganised state of the lungs, while the liver and other viscera have been perfectly healthy in their structure. To my mind, this is no proof of the pulmonary disease being a primary one: mere *disorder* of the abdominal viscera is capable of inducing *disease* of the lungs—this, once established, can seldom or never be relieved, but the viscera which gave rise to the disease may resume their healthy functions. The profession must ever feel grateful to Mr. Abernethy for his valuable enquiries on the digestive organs: they not only have thrown great light on medical science in general, but have taught us to account, in a physiological manner, for the success of various remedies which were before used empirically. From a careful observation of these organs, we have been led to adopt a rational treatment, and, in some measure, to prevent the frequent occurrence of hydrocephalus: from the same source, I trust, experience will derive the cheering hope of being able to lessen the ravages of phthysis. Till these facts are confirmed or confuted by further experience, or morbid anatomy, it at least behoves every practitioner, when called to a case of supposed phthysis, to inquire whether there has been, or still exists, any disorder of the abdominal viscera. To correct their morbid actions should be his first care; by so doing, he will at all times alleviate the sufferings of the patient, and not unfrequently rescue him from death.

*August*, 1817. A. Z.

